# Regulation of the *let-7a-3* Promoter by NF-κB

**DOI:** 10.1371/journal.pone.0031240

**Published:** 2012-02-13

**Authors:** David J. Wang, Aster Legesse-Miller, Elizabeth L. Johnson, Hilary A. Coller

**Affiliations:** Department of Molecular Biology, Princeton University, Princeton, New Jersey, United States of America; Tulane University Health Sciences Center, United States of America

## Abstract

Changes in microRNA expression have been linked to a wide array of pathological states. However, little is known about the regulation of microRNA expression. The *let-7* microRNA is a tumor suppressor that inhibits cellular proliferation and promotes differentiation, and is frequently lost in tumors. We investigated the transcriptional regulation of two *let-7* family members, *let-7a-3* and *let-7b*, which form a microRNA cluster and are located 864 bp apart on chromosome 22q13.31. Previous reports present conflicting data on the role of the NF-κB transcription factor in regulating *let-7*. We cloned three fragments upstream of the *let-7a-3/let-7b* miRNA genomic region into a plasmid containing a luciferase reporter gene. Ectopic expression of subunits of NF-κB (p50 or p65/RelA) significantly increased luciferase activity in HeLa, 293, 293T and 3T3 cells, indicating that the *let-7a-3/let-7b* promoter is highly responsive to NF-κB. Mutation of a putative NF-κB binding site at bp −833 reduced basal promoter activity and decreased promoter activity in the presence of p50 or p65 overexpression. Mutation of a second putative binding site, at bp −947 also decreased promoter activity basally and in response to p65 induction, indicating that both sites contribute to NF-κB responsiveness. While the levels of the endogenous primary *let-7a* and *let-7b* transcript were induced in response to NF-κB overexpression in 293T cells, the levels of fully processed, mature *let-7a* and *let-7b* miRNAs did not increase. Instead, levels of Lin-28B, a protein that blocks *let-7* maturation, were induced by NF-κB. Increased Lin-28B levels could contribute to the lack of an increase in mature *let-7a* and *let-7b*. Our results suggest that the final biological outcome of NF-κB activation on *let-7* expression may vary depending upon the cellular context. We discuss our results in the context of NF-κB activity in repressing self-renewal and promoting differentiation.

## Introduction

miRNAs are non-coding, single-stranded, conserved RNAs of ∼22 nucleotides that function as gene regulators in both animals and plants [1]. miRNAs have been discovered to play a central role in a wide variety of biological processes. They are initially transcribed as primary transcripts (pri-miRNAs) and then cleaved by the RNase III enzyme Drosha into 70- to 100-nt hairpin-shaped precursors [2], [3]. These pre-miRNAs are exported into the cytoplasm and processed by the RNase III enzyme Dicer to their mature form. Fully-processed miRNAs negatively regulate their targets by binding to partially complementary sequences in the 3′ UTR of target transcripts [4], [5], leading to transcript instability or inhibition of translation [6], [7].

The *let-7* microRNA was discovered in a chromosomal location that affects terminal differentiation of seam cells in *C. elegans* [5]. In humans and mice, there is an association between *let-7*, cell cycle, and differentiation. Inhibition of *let-7* in A549 lung cancer cells increases cell proliferation rates, whereas *let-7* overexpression blocks cell-cycle progression [8], [9]. Overexpression of *let-7* in primary human fibroblasts results in reduced cell proliferation and an accumulation of cells in the G2/M phase of the cell cycle [10]. In *C. elegans*, mice and humans, *let-7* expression is barely detectable in embryonic stages but increases after differentiation and in mature tissue [11], [12], [13]. The *let-7* family of miRNAs is consistently down-regulated in lung and colon cancers [8], [14], [15]. In lung cancers, low levels of *let-7* correlate with shorter survival after resection [8], [14], [16]. Low levels of *let-7* are found in some stem cell populations, and high expression of a *let-7* target gene has been used to enrich for stem cells from a mouse mammary epithelial cell line [17].

*Let-7* family members can be regulated transcriptionally or post-transcriptionally. As one example of transcriptional activation, MYC activation results in widespread repression of miRNA expression, including *let-7* family members [18]. Post-transcriptional regulation can be mediated by Lin28B, which blocks the maturation of primary-*let-7* (*pri-let-7*) in embryonic stem cells [19], [20] by causing 3′ uridylation of *pre-let-7*, thus rendering the transcript resistant to Dicer processing [21]. In addition, the KSRP RNA binding protein and hnRNP A1 have also been reported to promote and inhibit the processing of *pri-let-7*, respectively [22], [23].

The NF-κB family of transcription factors is induced by numerous stimuli including growth factors, DNA damaging agents, cytokines, oxidants, and viral and bacterial pathogens [24], [25]. In response to activation, NF-κB family members translocate to the nucleus, and regulate transcription of over 400 effector genes involved in immunoregulation, growth regulation, inflammation, carcinogenesis and apoptosis [26], [27]. The signaling response involves homo or heterodimerization of five members of the NF-κB family: p50 and its precursor p105 (NF-κB1), p52 and its precursor p100 (NF-κB2), p65 (RelA), c-Rel and RelB. The p65, RelB and c-Rel components contain transcriptional activation domains necessary for positive regulation of gene expression, while the p50 and p52 subunits can repress transcription by themselves or activate transcription in a complex with proteins that contain transcriptional activation domains [26]. NF-κB dimers bind promoters and enhancer regions containing the consensus sequence 5′ GGGRNWYCC 3′, where N is any base, R is a purine, W is an adenine or thymine and Y is a pyrimidine [28].

Iliopoulos and colleagues reported that transient induction of the Src oncogene in non-transformed human mammary epithelial cells (MCF10A) results in production of the cytokine interleukin-6 (IL-6), which drives and maintains cells in a transformed state [29]. This epigenetic switch is mediated by the transcription factor NF-κB, which then directly activates Lin28 transcription leading to rapid reduction in mature *let-7* levels. Since *let-7* directly inhibits IL-6 expression, levels of IL-6 increase. This further activates NF-κB, leading to a positive feedback loop.

Whereas Iliopoulos and colleagues found that NF-κB directly activates Lin28 and thereby reduces *let-7* levels, Garzon and colleagues report that NF-κB activates *let-7* during granulocytic differentiation of NB4 cells induced by all-trans-retinoic acid (ATRA) [30]. This suggests that the final biological outcome of NF-κB activation on *let-7* expression may vary depending upon the cellular context. We discovered that *let-7* is regulated during the transition from proliferation to quiescence [10] and wanted to explore a potential role for NF-κB in this process. In this report, we show that the *let-7a-3* promoter is positively regulated by NF-κB subunits in three different cell lines. Introduction of NF-κB subunits also resulted in increased expression of *pri-let-7a* and *pri-let-7b*, but did not result in a corresponding induction of the processed *let-7a* or *let-7b*. This could reflect, in part, an NF-κB-induced increase in the levels of Lin28B.

## Methods

### Computational analysis of the *let-7a-3* promoter

The DNA sequence of the *let-7a-3* promoter was analyzed with PROMO. The PROMO software identifies putative transcription factor binding sites with weight matrices representing consensus recognition sequences for different transcription factors as defined in the TRANSFAC database [31], [32]. NF-κB binding sites upstream of the *let-7a-3* start site were identified.

### Plasmids and promoter reporter constructs

Promoter regions were amplified from human genomic DNA using the following primer sets: 1 Kb 5′ ggtaccggacctcactctgctgcccccttggctgtgtgacatccagg and 1 Kb 3′ aagcttggtgccgatgggactccgtggcttc; 1.5 Kb 5′ gccggtacctagacctttcaagtccacttgggcatggggagctgagag and 1.5 Kb 3′ tggagatctgggcagtcggtcttggtgccgatgggactccgtggcttc; 3 Kb 5′ ggcggtaccctggcctctcctgtcacctagccaccgggc and 3 Kb 3′ tggagatctaaagggcagtcggtcttggt. The PCR products were cloned into the promoterless vector pGL3 Basic (Promega, Madison, Wisconsin) using KpnI/HindIII and KpnI/BglII restriction sites to generate pGL3-1Kb, pGL3-1.5Kb and pGL3-3Kb. Site-directed mutagenesis was performed with the QuikChange Kit (Stratagene, La Jolla, California) using the pGL3-1Kb vector as a template. Mutations were introduced in the NF-κB recognition site at −833 to generate two distinct mutant plasmids. In the first mutant plasmid (pGL3-1Kb-m1), the original sequence GGGGAGCCCC was changed to GGGCAGAACC by introducing three nucleotide substitutions using primers: 5′ ttcccctgctagggcagaaccgaggccctctca and 3′ tgagagggcctcggttctgccctagcaggggaa. In the second mutant plasmid (pGL3-pGL3-1Kb-m2), the sequence was changed to GAGCCCC, thus introducing a 3-bp deletion using primers: 5′ ccacgttcccctgctagagccccga and 3′ tcggggctctagcaggggaacgtgg. The NF-κB recognition site at −947 was also mutated (pGL3-1Kb-m3) using site-directed mutagenesis. The sequence was changed from 5′ AGCTTTTCCCC to 5′ ATTTCCCC using primers: 5′ gtatctgccccctcatttccccaggaaggt and 3′ accttcctggggaaatgagggggcagatac. pCMV2 vectors containing no insert, or the p50 or p65 subunits of NF-κB cloned downstream of the CMV promoter, were a generous gift of the Guttridge Lab, Ohio State University [30].

### Immunoblot analysis

HeLa or HEK293T cells were transfected with an empty vector control, a plasmid encoding p50, or a plasmid encoding p65 using Lipofectamine 2000 (Invitrogen, Carlsbad, California) according to the manufacturer's instruction. Cells were lysed in RIPA buffer containing protease and phosphatase inhibitors [10 mM NaPO4 pH 7.2, 0.3 M NaCl, 0.1% SDS, 1% NP40, 1% Na deoxycholate, 2 mM EDTA, protease inhibitor cocktail (Roche, Basel, Switzerland)]. Protein concentration was determined by the Lowry method using the Bio-Rad DC Protein Assay Kit II (Bio-Rad, Hercules, California) as described by the manufacturer. Equal amounts of total cellular proteins were resolved on SDS-PAGE and electro-transferred onto a PVDF membrane, which was then incubated with an antibody to p50 (Cell Signaling, Beverly, MA, 1∶1000 dilution), p65 (Cell Signaling, 1∶1000), Lin28B (Cell Signaling 1∶1000) or Flag (Sigma-Aldrich, St. Louis, MO, 1∶2000). Secondary antibodies conjugated with horseradish peroxidase (GE Healthcare, Little Chalfont, United Kingdom,1∶3000 dilution) and enhanced chemiluminescence (Pierce, Thermo Fisher Scientific, Waltham, Massachusetts) were used to detect the antigen. Membranes were stripped using Restore Western Blot Stripping Buffer (Thermo Fisher Scientific) and immunoblotted with GAPDH (Abcam, Cambridge, Massachusetts, 1∶5000 dilution) as a loading control.

### Luciferase reporter assays

Promoter activity was determined by co-transfection of the pGL3 promoter reporter with a plasmid designed to serve as a control for transfection efficiency, pRL-CMV (Renilla luciferase plasmid, Promega, Madison, Wisconsin), into four cell types. HEK293 (generously provided by the Flint laboratory, Princeton University), HEK293T (ATCC, Manassas, Virginia), NIH3T3 (ATCC) and HeLa (ATCC) cells were grown in DMEM (Invitrogen-GIBCO, Carlsbad, California) supplemented with 10% fetal bovine serum (FBS, Atlanta Biologicals, Lawrenceville, Georgia). Cells were grown to a cell density of 60–70% in 12-well dishes and then transiently transfected with 2 µg of experimental firefly luciferase plasmids, 0.2 µg of pRL-CMV, and 2 µg of empty vector plasmids or plasmids encoding the p50 or p65 NF-κB subunits using Lipofectamine 2000 (Invitrogen). Twenty-four hours post-transfection, cells were harvested and luciferase activity was measured using a GloMax™ 96-well Microplate Luminometer (Promega). The ratio of firefly to Renilla luciferase activity was determined.

### Transfection of NF-κB subunits

Lipofectamine 2000 (Invitrogen) was used to transfect 24 µg of plasmids encoding the NF-κB subunits p50, p65 or the pRL-CMV control vector into HEK293T cells according to the manufacturer's protocol. Media was replaced 4 hours after transfection and RNA was collected using TRIzol (Invitrogen) 24 hours after the start of the transfection.

### Real time PCR

To monitor levels of processed *let-7a* and *let-7b*, real-time PCR was performed with stem-loop primers and probes designed specifically to detect *let-7a* or *let-7b* (Applied Biosystems, Carlsbad, California). Total RNA was isolated using TRIzol Plus RNA purification system (Ambion, Life Technologies, Grand Island, New York). miRNA abundance was measured by real time PCR on an Applied Biosystems 7900HT Sequence Detection System using TaqMan microRNA assays according to the manufacturer's protocol (Applied Biosystems). The standard curve method was used to quantify unknown miRNA abundance and the threshold cycle (C_T_) was defined as the fractional cycle number at which the fluorescence passed a fixed threshold. The ratio of the amount of miRNA to the amount of a U6 small nuclear RNA control was determined for each sample.

To detect *pri-let-7* primary transcript, primers and probes provided with the TaqMan Pri-miRNA Assays (Applied Biosystems) were used. Two µg of RNA were reverse transcribed with the High Capacity RNA-to-cDNA Kit (Applied Biosystems). Standard curve dilutions of the cDNAs were prepared (1∶10 to 1∶10000) and real-time PCR was performed as described above. The ratio between fluorescence from *pri-let-7* detection and that of the control housekeeping gene β-actin, which was measured using Taqman β-actin Gene Expression Assays, was determined.

To detect Lin28B mRNA, RNA was reverse-transcribed with the High Capacity RNA-to-cDNA Kit (Applied Biosystems). Probes and primers were designed using the Integrated DNA Technologies PrimeTime qPCR Assay Design Tool. Real-time PCR was performed with the following mix: 25 µL Universal 2× Master Mix, 5 µL cDNA template, 3.3 µL primers (2 µM), 2.5 µL probe (2 µM) and 10.9 µL dH_2_O. Lin28B fluorescence was normalized to the β-actin control as mentioned above.

## Results

### The *let-7a-3* promoter is induced by NF-κB subunits

**Figure 1:**
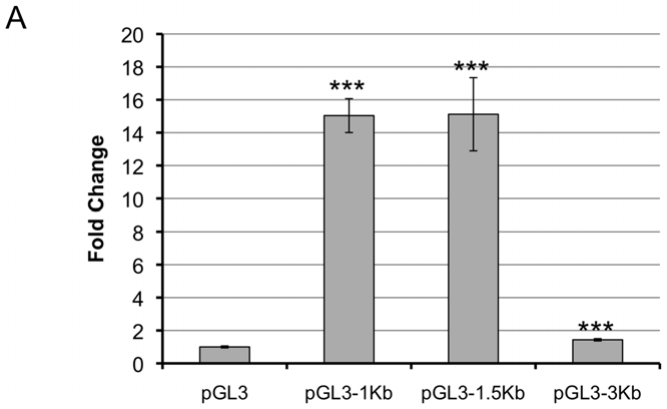
The upstream genomic region of *let-7a-3/b* microRNAs confers transcriptional activity. HEK293T cells were transfected with pGL3, pGL3-1Kb, pGL3-1.5Kb or pGL3-3Kb. Cells were collected 48 hours post transfection and luciferase activity was determined. Data are from two biological replicates, each with two technical replicates. Mean values are indicated and error bars designate the standard deviation.

In order to determine the minimum required region for the *let-7a-3/let-7b* microRNA cluster, the sequences 1 kb, 1.5 kb and 3 kb upstream of the *let-7a-3* miRNA were cloned into the pGL3 vector upstream of the gene encoding firefly luciferase to generate vectors pGL3-1Kb, pGL3-1.5Kb and pGL3-3Kb, and promoter activity was monitored. HEK293T cells were transfected with *let-7* promoter luciferase reporters or control plasmids along with a normalization plasmid for transfection efficiency expressing Renilla luciferase. Protein lysates collected 48 hours post-transfection were analyzed for the ratio of firefly to Renilla luciferase activity. Fold-change was determined with respect to pGL3 activity. The *let-7* promoter plasmids pGL3-1Kb and pGL3-1.5Kb produced significantly higher luciferase activity than the control plasmid (*p*<0.0001). Thus, even just 1 kb of the *let-7a-3* promoter was sufficient to induce promoter activity without the introduction of other factors (Figure 1). Promoter activity was lower for the pGL3-3Kb plasmid suggesting that there may be repressive elements between 1.5 kb and 3 kb upstream from the *let-7a-3* promoter.

**Figure 2:**
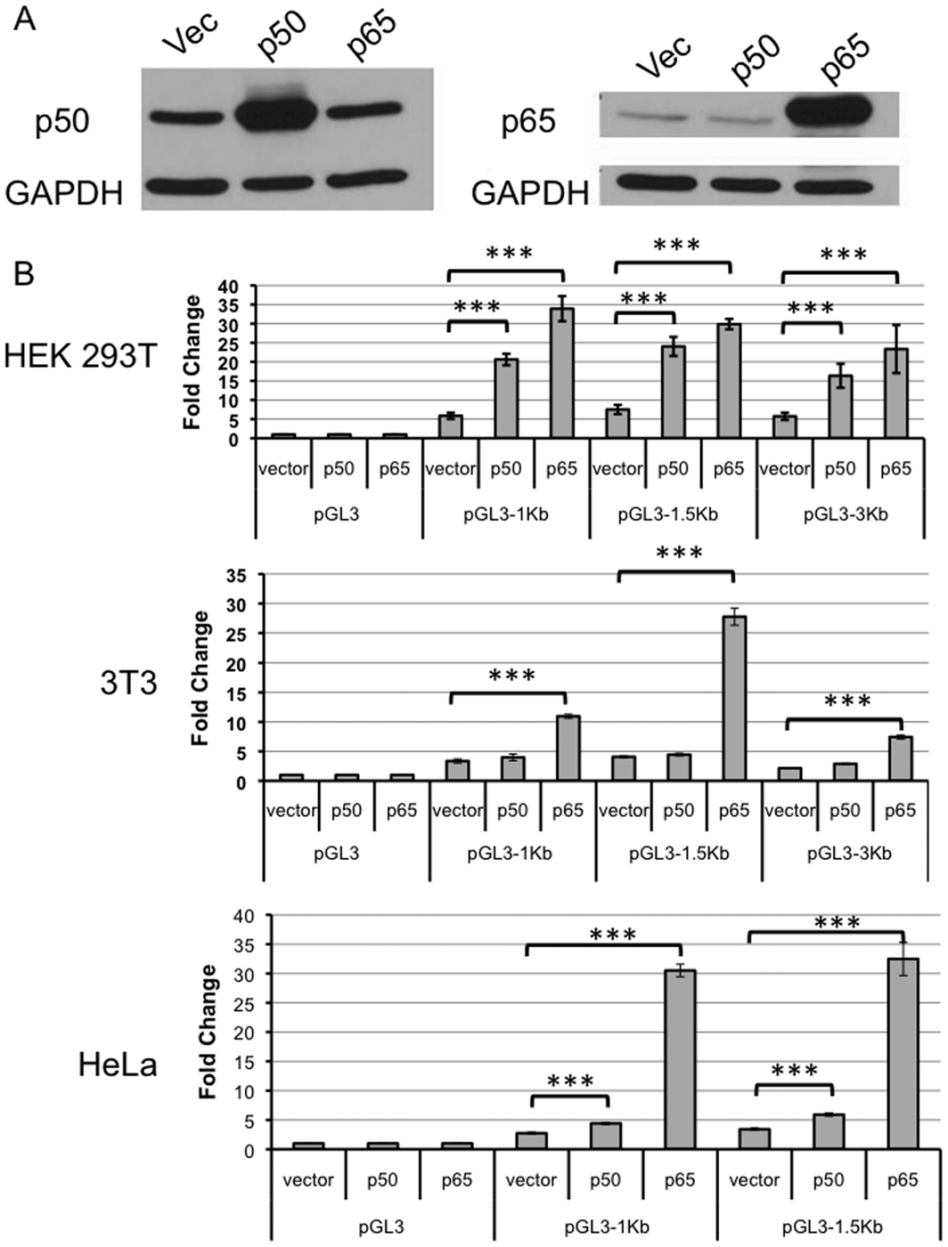
The *let-7a-3* promoter is regulated by overexpression of NF-κB transcription factor subunits p50 or p65. A. Transfection of plasmids expressing p50 or p65 results in increased levels of the encoded protein. HeLa cells were transfected with an empty vector or vectors expressing NF-κB subunits p50 or p65. Protein lysates were collected 48 hours post-transfection and levels of the encoded protein were determined with immunoblotting. GAPDH levels were monitored as a loading control. B. Transfection of plasmids expressing p50 or p65 results in increased promoter activity. Cells were co-transfected with a Renilla luciferase reporter; a control plasmid (vector) or plasmids that overexpress the p50 or p65 subunits of the NF-κB transcription factor; and the pGL3 vector or the pGL3 vector with 1 Kb, 1.5 Kb or 3 Kb of the *let-7a3* promoter region subcloned upstream of luciferase (repesented as pGL3-1Kb, pGL3-1.5Kb or pGL3-3Kb, respectively). Luciferase assays were performed to determine the amount of firefly and Renilla luciferase in each sample. For HEK293T cells, two technical replicates were performed for each of two biological replicates. For NIH 3T3 and HeLa, four technical replicates were performed for each of the two biological replicates. The ratio of firefly to Renilla luciferase was determined, and the fold change compared with the pGL3 vector was calculated. Mean values are plotted and error bars reflect standard deviations.

In order to investigate NF-κB responsiveness of the *let-7a-3* promoter, plasmids expressing p50 and p65 subunits of NF-κB were transfected into HeLa cells. Immunoblotting confirmed that the appropriate proteins were overexpressed (Figure 2A). NIH3T3 cells, HeLa cells, and HEK293T cells were transfected with a *let-7* promoter reporter plasmid, plasmids expressing constituents of the NF-κB transcription factor (p50 or p65) or an empty vector, and a normalization plasmid expressing Renilla luciferase (Figure 2B). Samples co-transfected with the p50 or p65 subunits exhibited significantly higher activity than samples co-transfected with the empty control plasmid. Luciferase activity was observed for the three different vectors containing the *let-7* promoter, indicating that the important elements were likely to be within the first 1 kb upstream of transcriptional start. In all three cell lines, a strong induction was observed with p65 transfection (*p*<0.0001). The extent to which p50 overexpression induced luciferase activity varied among the cell lines, with the highest induction in HEK293T cells and more modest induction in HeLa and 3T3 cells.

### Contribution of specific NF-κB binding sites to the NF-κB responsiveness of the *let-7a-3* promoter

**Figure 3:**
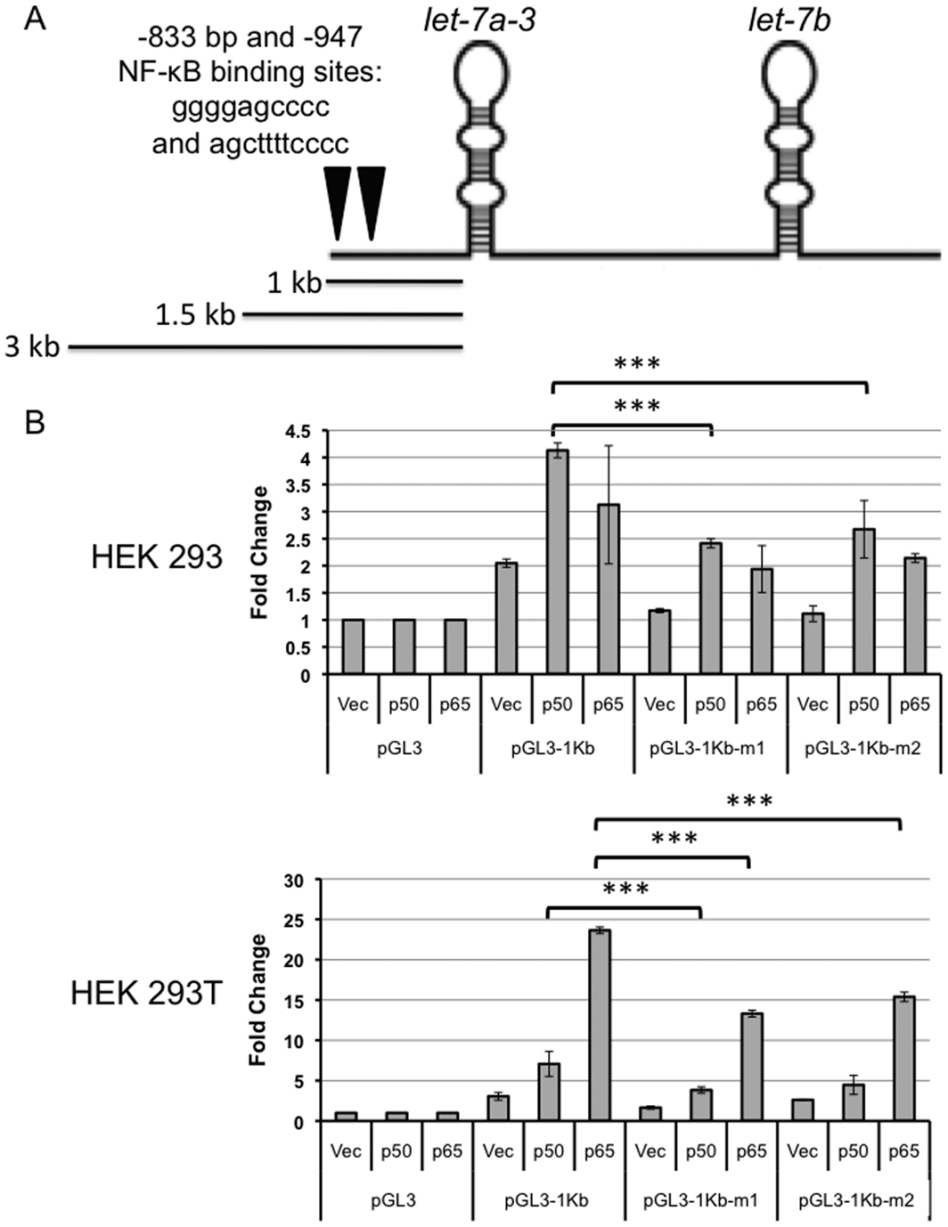
NF-κB regulates the *let-7a-3* promoter activity in part through a binding site at −833 bp. A. The genomic organization of the *let-7a-3/let-7b* miRNA cluster (chromosome 22) is shown. The location of the putative NF-κB binding site at −833 bp is indicated. Portions of the genomic region upstream of *let-7a-3* that were subcloned to create the 1 kb, 1.5 kb and 3 kb plasmids are also depicted. B. HEK293 or HEK293T cells were co-transfected with a Renilla luciferase reporter; a negative control expression plasmid (vector); and pGL3, pGL3-1Kb containing 1 Kb of wild-type *let-7a-3* promoter, or one of two clones of the same vector in which the putative NF-κB binding site at −833 bp was mutated (pGL3-1Kb-m1 and pGL3-1Kb-m2). Luciferase activity was monitored and the ratio of firefly to Renilla luciferase was determined. The fold-change compared with the pGL3 vector is plotted. Two biological replicates were performed and for each biological replicate, three technical replicates were analyzed. Mean values are indicated and error bars designate the standard deviation. Asterisks indicate *p*<0.001.

We analyzed the DNA sequence of the *let-7a-3* promoter region with PROMO software and discovered multiple NF-κB binding sites. These included a binding site that had been previously reported at −833 as well as binding sites −947 bp upstream, −649 bp upstream, −292 bp upstream and 243 bp upstream of the *let-7a-3* microRNA (Figure 3A). Garzon and colleagues reported that the NF-κB binding site at bp −833 is critical for transcription factor activity [30]. To test the role of this particular NF-κB binding site in NF-κB-mediated *let-7* promoter regulation, site-directed mutagenesis was performed on the putative NF-κB recognition site at −833 bp in the 1 kb plasmid to create two distinct mutant plasmids, pGL3-1Kb-m1 and pGL3-1Kb-m2. In the pGL3-1Kb-m1 plasmid, the original sequence, GGGGAGCCCC, was changed to GGGCAGAACC by introducing three nucleotide substitutions. In the pGL3-1Kb-m2 plasmid, the sequence was changed to GAGCCCC, thus introducing a 3-bp deletion.

Transfection of wild-type or mutant reporters, NF-κB subunits or an empty vector control, and a transfection efficiency normalization control Renilla plasmid was performed in HEK293 cells and HEK293T cells. Mutagenesis of the NF-κB recognition site at −833 bp reduced basal expression levels of the plasmids, that is, activity without p50 or p65 ectopic expression (Figure 3B). In HEK293 cells, luciferase levels in cells transfected with pGL3-1Kb-m1 or pGL3-1Kb-m2 were significantly lower than luciferase levels in cells transfected with pGL3-1Kb (*p*<0.0001). In HEK293T cells, luciferase levels for pGL3-1Kb-m1 were significantly lower than those in cells transfected with pGL3-1Kb. In HEK293T cells transfected with pGL3-1Kb-m2, luciferase activity was reduced but did not reach statistical significance. We conclude that in most cases mutation of the NF-κB binding site at −833 bp significantly reduced basal *let-7* promoter activity so that luciferase levels returned to a level close to, but in most cases distinguishable from, the levels in empty vector transfected cells. This suggests that the 1 Kb *let-7* promoter fragment also contains other sites that contribute to expression.

Mutagenesis of the −833 bp site also reduced but did not eliminate the responsiveness of the *let-7* promoter to overexpression of p50 or p65 in both HEK293 and HEK293T cells (Figure 3B). In HEK293 cells, for both pGL3-1Kb-m1 and pGL3-1Kb-m2, luciferase levels in cells transfected with p50 were lower than in cells transfected with pGL3-1Kb and p50 (*p*<0.0001). For p65 transfection, luciferase levels decreased when pGL3-1Kb-m1 or pGL3-1Kb-m2 was transfected, but the decrease did not achieve statistical significance. In HEK293T cells, levels for pGL3-1Kb-m1 with p50 or p65 were lower than with pGL3-1Kb (*p*<0.0001 for p65; *p* = 0.0043 for p50), and levels for pGL3-1Kb-m2 were lower with p65 (*p*<0.0001), but did not reach statistical significance with p50 (*p* = 0.06). Expression levels did not return to baseline levels with either pGL3-1Kb-m1 or pGL3-1Kb-m2 in the presence of p65 or p50. These findings confirm the importance of NF-κB binding to this particular recognition site at −833 bp for basal *let-7* promoter activity and the promoter's responsiveness to ectopically expressed NF-κB subunits. They also indicate further that there are likely to be other important regulators of *let-7* expression in addition to this particular binding site.

**Figure 4:**
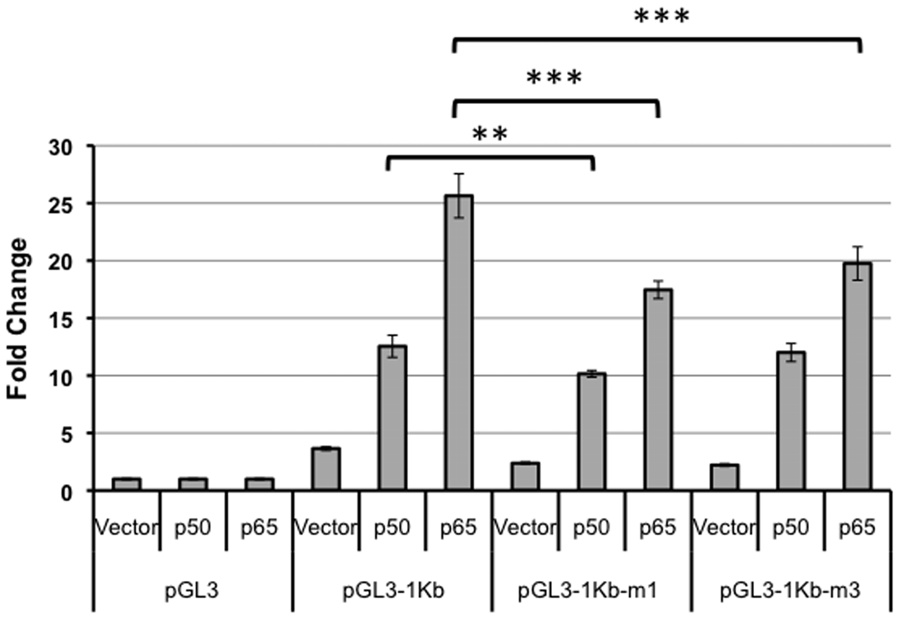
The NF-κB binding site at bp −947 also regulates *let-7a-3* promoter activity. HEK293T cells were co-transfected with plasmids containing either 1 kb of the wild-type *let-7a-3/b* promoter or one of two plasmids with mutated NF-κB recognition sites, one at bp −833 (pGL3-1Kb-m1) and one at bp −947 (pGL3-1Kb-m3), and plasmids containing NF-κB subunits. Normalization control pRenilla was also transfected. Luciferase activity was monitored and the ratio of firefly to Renilla luciferase was determined. The fold-change compared with the pGL3 vector is plotted. Two biological replicates were performed and for each biological replicate, three technical replicates were analyzed. Mean values are indicated and error bars designate the standard deviation.

In order to assess whether NF-κB activation of the *let-7a-3* promoter proceeds exclusively through the NF-κB recognition site at −833 bp, or whether other putative binding sites might also contribute to NF-κB activity, we extended our analysis to an additional putative NF-κB responsive element within the promoter at bp −947. We performed site-directed mutagenesis on the wild-type sequence 5′ AGCTTTTCCCC 3′ and converted it to 5′ ATTTCCCC 3′ to form pGL3-1Kb-m3. The pGL3-1Kb, pGL3-1Kb-m1 or pGL3-1Kb-m3 plasmids were co-transfected into HEK293T cells along with plasmids containing p50 or p65 subunits or no insert and luciferase activity was monitored. Transfection with either pGL3-1Kb-m1 or pGL3-1Kb-m3 resulted in significantly reduced basal activity compared to transfection with the wildtype pGL3-1Kb plasmid (*p*<0.0001) (Figure 4). Both pGL3-1Kb-m1 and pGL3-1Kb-m3 also resulted in reduced luciferase activity with p65 overexpression (*p*<0.0001). Cells transfected with pGL3-1Kb-m1 exhibited a larger decrease in luciferase activity in the presence of p50 than cells transfected with pGL3-1Kb-m3 (*p*<0.0001) and a larger decrease in luciferase activity in the presence of p65 than cells transfected with pGL3-1Kb-m3 (*p*<0.05). Thus, both the −833 and −947 sites are expected to contribute to NF-κB-regulated induction of the *let-7a-3/b* promoter, with the −833 bp site likely having a larger contribution to NF-κB responsiveness than the −947 site.

### NF-κB induces expression from the *let-7a/b* endogenous promoter but does not result in elevated levels of mature *let-7a* and *let-7b* transcripts

**Figure 5:**
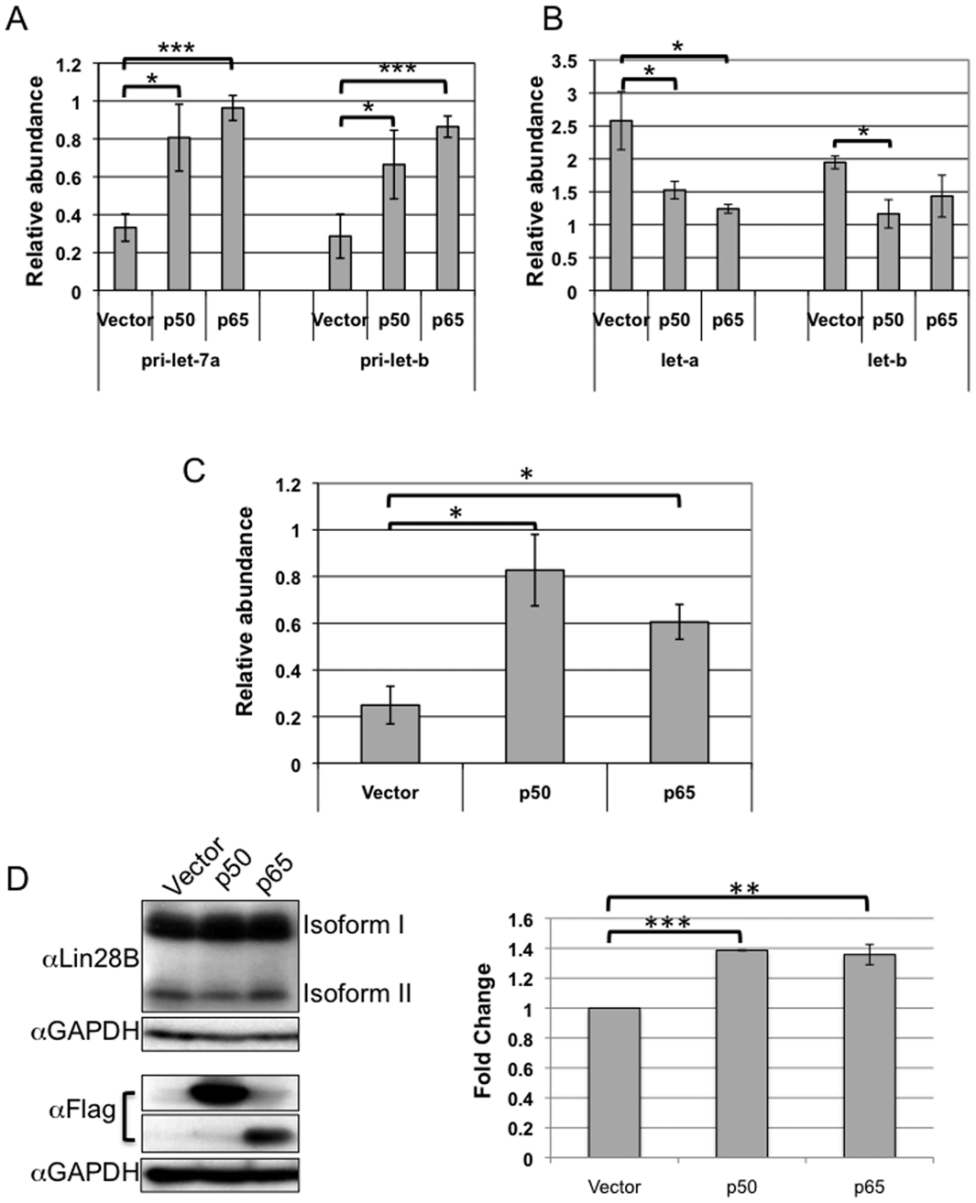
NF-κB induces *pri-let-7a/b* but not fully processed *let-7a/b*, possibly because of an induction of Lin28B. A. *Pri-let-7a-3* and *pri-let-7b* are induced by p65 and p50 in 293T cells. 293T cells were transfected with an empty vector or a vector expressing NF-κB subunits p50 or p65. Real-time PCR was performed for *pri-let-7a-3* and *pri-let-7b*. Data were normalized to β-actin. The data represent four biological replicates each performed in triplicate. Mean values are shown and error bars indicate standard error. One asterisk indicates *p*<0.05; three asterisks indicate *p*<0.001. B. Mature *let-7a* and *let-7b* are not induced by p50 or p65 in HEK293T cells. HEK293T cells were transfected with an empty vector or a vector expressing NF-κB subunits p50 or p65. Real-time PCR was performed and the ratio of *let-7a* or *let-7b* to U6 small nuclear RNA was determined. Error bars indicate standard error and represent an average from two biological replicates each performed in triplicate. Asterisk indicates *p*<0.05. C. Lin28B levels increase in HEK293T cells transfected with NF-κB subunits. HEK293T cells were transfected with an empty vector or a vector expressing NF-κB subunits p50 or p65. Real-time PCR was performed and the Lin28B to β-actin expression was determined. Error bars indicate standard error and represent an average from three biological replicates each performed in triplicate. Asterisk indicates *p*<0.05. D. Lin28B protein levels increase modestly in HEK293T cells expressing NF-κB subunits. Lin28B, p50 and p65 protein levels were monitored by Western blotting in lysates prepared from HEK293T cells transfected with an empty vector, or vectors expressing p50 or p65 (left). Anti-flag antibodies were used to measure exogenously expressed p50 and p65. Image J was used to quantify Lin28B protein levels for each sample in three independent experiments, and the fold change of Lin28B isoform I and II combined from samples expressing p50 or p65 to the empty control vector is plotted (right). Error bars represent standard error. Two asterisks indicates *p*<0.01. Three asterisks indicates *p*<0.001.

We tested whether transfection of NF-κB subunits results in an induction of endogenous mature *let-7a* or *let-7b*. Empty vector plasmids or plasmids expressing p50 or p65 were transfected into 293T cells and levels of *pri-let-7a* and *pri-let-7b* were monitored with real-time PCR using TaqMan pri-miRNA assays. *Pri-let-7a* and *pri-let-7b* transcript levels in each sample were normalized to β-actin levels in the same sample as a control. Both p65 and p50 overexpression resulted in a statistically significant increase in both *pri-let-7a* and *pri-let-7b* (Figure 5A). Thus, not only do NF-κB subunits activate luciferase activity when the luciferase gene is placed downstream of *let-7* promoter sequences, but the endogenous *let-7a3/let-7b* genes are also activated by NF-κB subunits in HEK293T cells.

We then tested whether introduction of NF-κB subunits results in an increase in mature *let-7a* or *let-7b* miRNA abundance. p50 or p65 were transfected into 293T cells and levels of the processed *let-7a* or *let-7b* were monitored with real-time PCR using TaqMan MicroRNA assays. U6 was used as a normalization control. Despite the fact that overexpression of p65 and p50 resulted in an induction of *pri-let-7a3* and *pri-let-7b*, we detected a significant decrease in mature *let-7a* and a moderate reduction of mature *let-7b* miRNA levels in p50 or p65 overexpressing cells (Figure 5B).

### NF-κB induces Lin28B

Our results indicate that while levels of the *pri-let-7a3/let-7b* transcript are induced in response to NF-κB subunits, the final processed form of the *let-7a3/let-7b* microRNAs are not. Previous studies had indicated that Lin28 is induced in response to NF-κB in Src-transformed MCF10A cells [29]. Increased Lin28B, a protein that prevents the processing of *let-7* pri-miRNAs, could potentially explain the fact that the increased levels of the pri-microRNAs did not result in elevated levels of the mature microRNA. We transfected p50, p65 or a control vector into 293T cells and monitored Lin28B levels with gene-specific primers and probes using real-time PCR and normalized to a β-actin control. Lin28B transcript levels increased in response to introduction of either p50 or p65 (Figure 5C). Overexpression of p50 or p65 resulted in a modest increase in Lin28B protein levels (Figure 5D). Increased Lin28B may partially explain the lack of up-regulation of the mature microRNA in response to p50 or p65 overexpression.

## Discussion

In order to better understand the regulation of the *let-7* miRNA family, we investigated one particular genomic region that contains *let-7a-3* and *let-7b*. We discovered that the *let-7a-3* promoter is responsive to NF-κB subunits p50 and p65. Similarly, Garzon and colleagues discovered that ATRA treatment of acute promeylocytic leukemia cell lines resulted in induction of *let-7a* and *let-7b*, and that NF-κB inhibitors abrogated this induction [30]. Using chromatin immunoprecipitation, Garzon and colleagues did not detect a significant amount of binding of p65 to the NF-κB motif within the *let-7* promoter and concluded that p50/p50 homodimers are likely responsible for the NF-κB-responsiveness of the promoter. Their experiments with small interfering RNAs against p50 and p65, however, suggested that p65 binding sites might be present. In our study, we discovered that luciferase activity is induced by overexpression of either p50 or p65 in 293, 293T, HeLa and 3T3 cells containing a reporter with the *let-7a-3* promoter was cloned upstream of the coding region for luciferase. In our experiments, induction with p65 was stronger than p50. Since p65 contains a transactivation domain and p50 does not, cell-type specificity in the activity of transfected p50 may reflect differences in the presence of endogenous co-factors like p65.

Garzon and colleagues also investigated the importance of the NF-κB binding site at −833 and concluded that this particular site is mostly responsible for NF-κB responsiveness [30]. This particular binding site was the only one to which p50 bound in their experiments. Further, in their studies, site-directed mutagenesis of this site essentially eliminated NF-κB-induced expression. While our data also support the conclusion that the recognition site at bp −833 is important under basal conditions and upon introduction of NF-κB subunits, in our studies, abrogation of the −833 bp site reduced but did not eliminate NF-κB responsiveness. We discovered that a second site at bp −947 also likely contributes to the induction of *let-7* upon NF-κB activation.

Overexpression of p65 resulted in an induction of *pri-let7a-3* and *pri-let-7b*, indicating that these two miRNAs likely form a polycistron. However, levels of the final processed *let-7a* or *let-7b* were not induced as assessed by real-time PCR. One potential explanation for the lack of final microRNA is the induction of Lin28, a potent regulator of *let-7* microRNA biogenesis, that recruits a terminal transferase Tut4 to add terminal uridines to *let-7* miRNAs, resulting in their degradation [33]. In 293T cells, in accord with previously reported results in Src-transformed MCF10A cells [29], Lin28B transcript and protein levels were induced by p50 or p65, although the protein level induction was modest. Increased Lin28B levels in response to NF-κB subunits could explain the lack of an increase in mature *let-7a* and *let-7b*.

NF-κB can have different effects depending on the cell type and cellular context, in some cases promoting proliferation [25], [27], [34], [35], and in other instances causing cell cycle arrest [36], [37]. This duality could potentially reflect, in part, the effect of NF-κB on *let-7*. Under certain circumstances, NF-κB activation might result in increased *let-7* transcription and in higher levels of processed *let-7*, as observed by Garzon and colleagues in NB4 cells [30]. Since *let-7* can act as a mediator of cell cycle exit, and is associated with a commitment to differentiation rather than self-renewal, *let-7* induction by NF-κB could be part of the mechanism by which NF-κB contributes to cell cycle exit. This could be advantageous in response to genotoxic damage, for instance, by providing extra time during G2/M arrest for repairing damage before resuming the cell cycle [10].

In other situations, NF-κB-mediated activation of transcription of the pri-miRNA for *let-7a-3/let-7b* could inhibit cell cycle progression in a delayed and regulated manner. As an analogy, the core embryonic stem cell transcription factors—Oct4, Nanog, Sox2 and Tcf3—promote the transcription of the miRNA *let-7g* and Lin28 [38]. When stem cells receive a differentiation signal, Lin28B-mediated inhibition is released and mature *let-7* starts to accumulate. By transcribing, but initially repressing *let-7*, embryonic stem cells are poised for rapid and efficient cellular differentiation. Similarly, our results suggest that NF-κB activates the *let-7a-3* promoter and Lin28B in HEK293T cells. While mature *let-7a* and *let-7b* do not accumulate immediately, the cells could be poised for elevated *let-7* activity if Lin28B or other processing factors were later inhibited.

NF-κB activation can also result in lower *let-7* levels, thus increased cellular inflammation and increased tumorigenesis [29]. These results, taken together with ours, suggest a possible mechanism for the dual role of NF-κB in both inhibiting and promoting tumorigenesis. In cells expressing Lin28B or other factors that inhibit *pri-let-7* processing, NF-κB activation would result in unchanged or reduced *let-7* levels. In committed cells that lack the critical regulatory factors, for example hematopoietic cells, NF-κB activity results in the induction of mature *let-7*. This could allow for removal of damaged cells or provide more time for repair. In this hypothetical model Lin28 or other miRNA biogenesis regulatory molecules might contribute to the determination of the ultimate functional effect of NF-κB.
